# Relation between single serum progesterone assay and viability of the first trimester pregnancy

**DOI:** 10.1186/2193-1801-1-80

**Published:** 2012-12-27

**Authors:** Ibrahim A Abdelazim, Amro Abo Elezz, Mohamed Elsherbiny

**Affiliations:** 1 Obstetrics & Gynecology, Ain Shams University, Cairo, Egypt and Ahmadi Hospital, Kuwait Oil Company, P.O.Box: 9758, Ahmadi, 61008 Kuwait; 2 Assistant Professor of Obstetrics & Gynaecology, Al-Azhar University, Cairo, Egypt; 3 Specialist of ultrasound, Fetal Care unit, Ain Shams University, Ciaro, Egypt; 4 Ahmadi Hospital-Kuwait Oil Company, Kragujevac, Kuwait

**Keywords:** Serum progesterone, Viability, First trimester pregnancy

## Abstract

This study was designed to detect the relation between serum progesterone and viability of pregnancy during the first trimester. Prospective study carried out in Al-Rashid Maternity and Ahmadi Kuwait oil company hospitals, over three years from February 2009 to February 2012. Two hundred and Sixty (260) pregnant women were hospitalized due to vaginal bleeding and/or abdominal pain during the first trimester of their pregnancies and were included in this study. Women included in this study were; sure of dates, conceived spontaneously with no history of infertility and had a positive serum pregnancy test. 2 ml blood samples were taken for women included in this study for serum progesterone assay. Women included in this study were followed by ultrasound for the viability of the pregnancy till the end of first trimester and the outcome of their pregnancy were recorded, while women with exogenous progesterone support or multiple pregnancies or suspected ectopic pregnancy or Hydatiform mole were excluded from this study. Data were collected and statistically analyzed to detect the relationship between serum progesterone level and viability of pregnancy during the first trimester. The mean age of the studied population was 32.7 ± 5.1 years, the mean gestational age at progesterone assay was 9.7 ± 0.5 week and by the end of the first trimester, women included in this study were classified according to the viability of their pregnancies into; viable pregnancy group 178 (68.5%) cases and non-viable pregnancy group (ended by miscarriage) 82 (31.5%) cases. The mean serum progesterone of the studied population was significantly high in viable pregnancy group (46.5 ± 7.4 ng/ml) compared to non-viable pregnancy group (9.9 ± 4.8 ng/ml), (p <0.05). In this study; 6.7% of viable pregnancies had serum progesterone level <10 ng/ ml, while 20.7% of non-viable pregnancies had serum progesterone level >10 ng/ml, the serum progesterone at cut off level 10 ng/ml was 79.3% sensitive to diagnose non-viable pregnancy and was 93.3% specific to diagnose viable pregnancy. Also, in this study; 1.1% of viable pregnancies had serum progesterone level <20 ng/ ml, while 4.8% of non-viable pregnancies had serum progesterone level >20 ng/ml, the serum progesterone at cut off level 20 ng/ml was 95.1% sensitive to diagnose non-viable pregnancy and was 98.9% specific to diagnose viable pregnancy. Serum progesterone is a reliable marker for early pregnancy failure and single assay of its serum level can differentiate between viable and non-viable pregnancies.

## Introduction

Ultrasound scanning is probably the best single diagnostic and prognostic test available for diagnosing early pregnancy failure. However, there were certain conditions where both sonographic and clinical findings were indeterminate or inconclusive (Elson et al. 2003).

Progesterone is a C-21 steroid hormone secreted by granulosa cells of the ovary. This hormone is important to promote endometrial decidualization by preparing the uterus for implantation of the blastocyst and to maintain the pregnancy (Hanita & Hanisah 2012). The physiological functions of progesterone include; inhibition of smooth muscle contractility and inhibition of immune responses like those involved in graft rejection (Hanita & Hanisah 2012).

Recent studies suggest that serum progesterone measured in early pregnancy is the most powerful single predictor of pregnancy outcome in natural conceptions (Elson et al. 2003). Few studies have attempted to use serum progesterone assay to predict the outcome of pregnancy in IVF/ICSI or in natural pregnancies and none has produced convincing conclusions (Homan et al. 2000). It is essential to study women after natural conceptions without exogenous progesterone support, when the relation between serum progesterone and viability of the first trimester pregnancy was evaluated (Vicdan & Zeki Isik 2001; Zainab Ali Abdulla Al 2000). So, this prospective study was designed to detect the relation between serum progesterone and viability of the pregnancy during the first trimester.

## Patients & methods

This study was carried out in Al-Rashid Maternity and Ahmadi Kuwait Oil Company hospitals, over 3 years from February 2009 to February 2012. Two hundred and Sixty (260) women were hospitalized due to vaginal bleeding and/or abdominal pain during the first trimester of their pregnancies and were included in this prospective study after informed consent and approval of the study protocol by institute ethical committee of both hospitals.

Data were collected from women included in this study by direct questioner to detect; age, parity, gestational age (calculated from the 1st day of LMP) and past history of early pregnancy miscarriages. Women included in the study were; sure of dates, conceived spontaneously with no history of infertility and had a positive serum pregnancy test.

2 ml blood samples were taken from women included in this study for serum progesterone assay and the samples were collected without anticoagulant in dry tubes. Serum was separated by centrifugation and stored at 2-8°C until hormonal assay. The assay principle combines an enzyme immunoassay competition method with final fluorescent detection. Women included in the study were examined by ultrasound for viability of the pregnancy and accordingly the results were classified into: viable and non-viable pregnancies. Those with inconclusive sonographic findings were re-examined by ultrasound again after two weeks and according to the findings they were reclassified into viable and non-viable pregnancies (anembryonic or missed miscarriage). Women included in this study were followed by ultrasound for the viability of the pregnancy till the end of first trimester and the outcome of their pregnancy were recorded, while women with exogenous progesterone support or multiple pregnancies or suspected ectopic pregnancy or Hydatiform mole were excluded from this study.

The ultrasound was done by an expert sonographer, who was blinded to the patients’ data, using Philips HD9 with 2D convex probe 4–9 MHz. Data were collected and statistically analyzed to detect the relationship between serum progesterone level and viability of the pregnancy during first trimester.

## Sample size justification

Using data of previous studies (Phipps et al. 2000; Hahlin et al. 1991), setting the type-1 error (α) at 0.05, the power (1-β) at 0.8 and assuming a 5% dropout rate, the number of participants needed to produce a statistically acceptable figure was more than two hundred women.

## Statistical analysis

Data were collected, tabulated then statistically analyzed using Statistical Package for Social Sciences (SPSS); computer software version (15). Numerical variables were presented as mean and standard deviation (±SD), while categorical variables were presented as number and percentage.

Chi-square test (X^2^) was used for comparison between groups as regard qualitative variables. A difference with p value <0.05 was considered statistically significant, otherwise it was insignificant. Sensitivity: is the proportional detection of individuals with the disease of interest in the population. Specificity: is the proportional detection of individuals without the disease of interest in the population.

## Results

Two hundred and Sixty (260) women were hospitalized due to vaginal bleeding and/or abdominal pain during first trimester of their pregnancy and were included in this study. The mean age of the studied population was 32.7 ± 5.1 years (ranged from 18–38 years), their mean parity was 4.2 ± 5.7 (ranged from 0–9) and the mean gestational age at progesterone assay was 9.7 ± 0.5 weeks (ranged from 7–11 weeks), (Table 1).

**Table 1:** **The characteristics of the studied population**

Variables	Mean ± SD	Range
Age (Year)	32.7 ± 5.1	18 - 38
Parity	4.2 ± 5.7	0 - 9
Gestational age at progesterone assay (Weeks)	9.7 ± 0.5	7 - 11

By the end of first trimester, women included in this study were classified according to the viability of their pregnancies into; viable pregnancy group 178 (68.5%) cases and non-viable pregnancy group (ended by miscarriages) 82 (31.5%) cases, (Table 2).

**Table 2:** **Classification of the studied population according to the viability of the pregnancy**

Ultrasound findings	Number (n)	Percentage (%)
Viable pregnancy group	178	68.5%
Non-viable pregnancy group	82	31.5%
Missed abortion	53	20.4%
Anembryonic (blighted ovum)	29	11.1%
Total number of cases	260	100%

The mean serum progesterone was significantly high in viable pregnancy group 46.5 ± 7.4 ng/ml (ranged from 18.7 - 86.3 ng/ml), compared with non-viable pregnancy group 9.9 ± 4.8 ng/ml (ranged from 1.67 - 26.2 ng/ml), (Chi-square test, p <0.05), (Table 3).

**Table 3:** **The relation between serum progesterone and viability of the pregnancy**

Pregnancy outcome	Number (%)	Serum progesterone (ng/ml)	Test used
		Mean ± SD (range)	P value (significance)
Viable Pregnancy group	178 (68.5%)	46.5 ± 7.4 (18.7 - 86.3)	Chi-square (X^2^)
Non-viable Pregnancy group	182 (31.5%)	9.9 ± 4.8 (1.67 - 26.2)	P <0.05 = 0.036
			(significant)

The relations between serum progesterone and maternal age or gestational age of the studied populations were statistically insignificant, also the relation between serum progesterone and past history of early miscarriage was statistically insignificant (Chi-square test; p >0.05), (Table 4).

**Table 4:** **The relation between serum progesterone and maternal age, gestational age or past history of early miscarriage**

Variables	Total number	Serum progesterone	Test used
	(n = 260)	(ng/ml)	P value
		Mean ± SD	(Significance)
Maternal age			Chi-square (X^2^)
>35 years old	142	24.62 ± 8.2	P >0.05 = 0.76
< 35 years old	118	18.52 ± 6.8	(Non-significant)
Past history of early miscarriage			Chi-square (X2)
Positive	48	12.26 ± 2.3	P >0.05 = 0.07
Negative	212	27.81 ± 5.7	(Non-significant)
Gestational age			Chi-square (X^2^)
>10 weeks gestation	77	16.27 ± 4.7	P >0.05 = 0.27
< 10 weeks gestation	183	26.45 ± 3.9	(Non-significant)

In this study; 6.7% of viable pregnancies had serum progesterone level <10 ng/ ml, while 20.7% of non-viable pregnancies had serum progesterone level >10 ng/ml, the serum progesterone at cut off level 10 ng/ml was 79.3% sensitive to diagnose non-viable pregnancy and was 93.3% specific to diagnose viable pregnancy. Also, in this study; 1.1% of viable pregnancies had serum progesterone level <20 ng/ ml, while 4.8% of non-viable pregnancies had serum progesterone level >20 ng/ml, the serum progesterone at cut off level 20 ng/ml was 95.1% sensitive to diagnose non-viable pregnancy and was 98.9% specific to diagnose viable pregnancy (Table 5).

**Table 5:** **Relations between serum progesterone cut off levels and viability of the pregnancy**

Variables	Viable	Non-viable
	Pregnancy group (Total number = 178)	Pregnancy group (Total number = 82)
Serum Progesterone at cut off level 10 ng/ml		
Number of cases with serum progesterone < 10 ng/ml (%)	12 (6.7%)	65 (79.3% = sensitivity)
Number of cases with serum progesterone > 10 ng/ml (%)	166 (93.3% = specificity)	17 (20.7%)
Serum Progesterone at cut off level 20 ng/ml		
Number of cases with serum progesterone < 20 ng/ml (%)	2 (1.1%)	78 (95.1% = sensitivity)
Number of cases with serum progesterone > 20 ng/ml (%)	176 (98.9% = specificity)	4 (4.8%)

## Discussion

Recent studies suggest that serum progesterone measured in early pregnancy is the most powerful single predictor of pregnancy outcome in natural conceptions (Elson et al. 2003; Zainab Ali Abdulla Al 2000; Phipps et al. 2000). So, this prospective study was designed to detect the relation between serum progesterone and viability of the pregnancy during the first trimester.

Two hundred and Sixty (260) women were hospitalized due to vaginal bleeding and/or abdominal pain during the first trimester of their pregnancies and were included in this prospective study. The mean age of the studied population was 32.7 ± 5.1 years, the mean gestational age at progesterone assay was 9.7 ± 0.5 week and by the end of the first trimester, women included in this study were classified according to the viability of their pregnancies into; viable pregnancy group 178 (68.5%) cases and non-viable pregnancy group (ended by miscarriages) 82 (31.5%) cases. The mean serum progesterone of the studied population was significantly high in viable pregnancy group (46.5 ± 7.4 ng/ml) compared to non-viable pregnancy group (9.9 ± 4.8 ng/ml).

Progesterone level and daily change in human chorionic gonadotropin (β-hCG) were determined in the serum of 307 patients with suspected ectopic pregnancy by Hahlin et al., and they found that 99% of the viable intrauterine pregnancies had serum progesterone more than 30 nmol/l (9.42 ng/ml; 1 nmol/1 = 0.314 ng/ml), whereas 75% of the ectopic pregnancy and 81% of the spontaneous abortions had serum progesterone less than 30 nmol/l (9.42 ng/ml), also, serum samples for progesterone, inhibin A, hCG, and urine beta-core hCG were collected from 220 women presented in the first trimester of pregnancy with complaints of pain, cramping, bleeding or spotting by Phipps and colleagues, to evaluate whether those biomarkers could predict viable and non-viable outcomes in pregnancy, and they concluded that serum progesterone was the most specific single biomarker for distinguishing viable from non-viable pregnancies (Hahlin et al. 1991; Phipps et al. 2000).

Although, Lijun & colleagues concluded that serum progesterone combined with β-hCG measurements, with a diagnostic accuracy of 85.7%, had the best prognostic reliability for predicting the outcome of threatened miscarriage compared to serum progesterone alone or β-hCG alone (Duan et al. 2011), Daily and colleagues found that the mean serum progesterone was significantly high for viable pregnancies (22.1 ng/ml) compared to non-viable pregnancies (10.1 ng/ml) and they concluded that a serum progesterone assay alone is predictive of pregnancy outcome specially during the first 8 weeks of gestation (Daily et al. 1994), also, Zainab Al Jufairi, found that serum progesterone level was significantly high in patients with viable pregnancies (20.48 ± 6.066 ng/ml) compared with patient with non-viable pregnancies ended by spontaneous abortion (7.78 ± 2.06 ng/ml) and she concluded that the serum progesterone alone is a reliable marker for prediction of early pregnancy failure (Zainab Ali Abdulla Al 2000).

The relations between serum progesterone and maternal age or gestational age of the studied population were statistically insignificant; also the relation between serum progesterone and past history of early miscarriage was statistically insignificant.

In this study; 6.7% of viable pregnancies had serum progesterone level <10 ng/ ml, while 20.7% of non-viable pregnancies had serum progesterone level >10 ng/ml, the serum progesterone at cut off level 10 ng/ml was 79.3% sensitive to diagnose non-viable pregnancy and was 93.3% specific to diagnose viable pregnancy. Also, in this study; 1.1% of viable pregnancies had serum progesterone level <20 ng/ ml, while 4.8% of non-viable pregnancies had serum progesterone level >20 ng/ml, the serum progesterone at cut off level 20 ng/ml was 95.1% sensitive to diagnose non-viable pregnancy and was 98.9% specific to diagnose viable pregnancy.

Ninety-five (95) pregnant women of 13 weeks or less were recruited as study group and fourteen (14) normal pregnant women were recruited as controls, to determine the role of serum progesterone as a marker of early pregnancy failure after single assay by Hanita and colleagues. They found that the serum progesterone levels were significantly lower in women with non-viable pregnancies compared with women with viable pregnancy (10.7 ng/ml versus 45.9 ng/ml; respectively). Hanita and colleagues, concluded that serum progesterone can be used as a marker for early pregnancy failure and at cut-off value of 32.7ng/ml, serum progesterone had 90% sensitivity with 75% NPV and 92% specificity with 97% PPV to diagnose early pregnancy failure (Hanita & Hanisah 2012).

Four hundred and eighty-nine (489) women presenting with singleton pregnancy, vaginal bleeding and/or abdominal pain in the first 18 weeks of pregnancy were included in a prospective comparative study was conducted by Al-Sebai et al., to assess the role of a single maternal serum progesterone measurement in the immediate diagnosis of early pregnancy failure and in the long term prognosis of fetal viability. They found that serum Progesterone levels were significantly lower in the non-continuing and tubal pregnancy groups compared to threatened-continuing groups and a cut-off level at 45 nmol/l (14.13 ng/ml) was found to differentiate between the viable pregnancies and the abnormal (non-continuing) pregnancies with 87.6% sensitivity and 87.5% specificity. Al-Sebai and colleagues concluded that a single serum progesterone measurement taken in early pregnancy is valuable in the immediate diagnosis of early pregnancy failure and the long term prognosis of viability (Al-Sebai et al. 2005).

Also, a prospective study was conducted by Ioannidis and colleagues, to investigate the relation between early (14 days after oocyte recovery) serum progesterone assay and pregnancy outcome in women undergoing IVF/ICSI and receiving rectal progesterone supplements. They found that the single progesterone assay on day 14 post-oocyte retrieval was significantly high in women with on-going pregnancies compared to women with an abnormal pregnancy. Ioannidis and colleagues concluded that single serum progesterone measurement could be a useful indicator of pregnancy outcome in women undergoing IVF/ICSI treatment (Ioannidis et al. 2005).

## Conclusion

Serum progesterone is a reliable marker for early pregnancy failure and single assay of its serum level can differentiate between viable and non-viable pregnancies.

## Abbreviations

ICSI: Intracytoplasmic sperm injection
IVF: In vitro fertilization
LMP: Last Menstrual period.
